# It’s All in the Interaction: Early Acquired Words Are Both Frequent and Highly Imageable

**DOI:** 10.1162/opmi_a_00130

**Published:** 2024-03-26

**Authors:** Joseph R. Coffey, Margarita Zeitlin, Jean Crawford, Jesse Snedeker

**Affiliations:** Department of Psychology, Harvard University

**Keywords:** vocabulary, frequency, imageability, CDI, acquisition, syntactic categories

## Abstract

Prior studies have found that children are more likely to learn words that are frequent in the input and highly imageable. Many theories of word learning, however, predict that these variables should interact, particularly early in development: frequency of a form is of little use if you cannot infer its meaning, and a concrete word cannot be acquired if you never hear it. The present study explores this interaction, how it changes over time and its relationship to syntactic category effects in children acquiring American English. We analyzed 1461 monolingual English-speaking children aged 1;4–2;6 from the MB-CDI norming study (Fenson et al., 1994). Word frequency was estimated from the CHILDES database, and imageability was measured using adult ratings. There was a strong over-additive interaction between frequency and imageability, such that children were more likely to learn a word if it was both highly imageable and very frequent. This interaction was larger in younger children than in older children. There were reliable differences between syntactic categories independent of frequency and imageability, which did not interact with age. These findings are consistent with theories in which children’s early words are acquired by mapping frequent word forms onto concrete, perceptually available referents, such that highly frequent items are only acquired if they are also imageable, and vice versa.

## INTRODUCTION

Language is an ability that is both universal and unique to humans. Despite the variability of their circumstances, almost all human children quickly become fluent speakers of the language (or languages) of their community. Between 12 and 30 months, American infants learn around 300 to 1200 words (Bates et al., 1994; Hart & Risley, 1995; Robinson & Mervis, 1999). While there is considerable variation in children’s vocabulary size (Bates et al., 1994; Braginsky et al., 2019) and the specific words that they learn (e.g., Mani & Ackermann, 2018; Oshima-Takane et al., 1996; Pine, 1995; Wallentin & Trecca, 2022) there is also a remarkable degree of consistency across children and languages in the kinds of words that are acquired early and those that are acquired later (Bornstein et al., 2004; Caselli et al., 1995; Fenson et al., 1994; Gentner et al., 2001; Sandhofer et al., 2000). A central question for developmental psychology is why some words are learned earlier than others. What are the characteristics of these early-learned words and what does this tell us about the process by which children learn them?

Prior research has identified three factors that are particularly strong predictors of which words children will acquire early. First, children are more likely to learn words that are more imageable, or easier to picture in one’s mind (Braginsky et al., 2019; Gentner, 1982; Gillette et al., 1999; Hansen, 2017; Hao et al., 2015; Ma et al., 2009; McDonough et al., 2011; Smolík, 2019). Second, they are more likely to learn words that are more frequent in their input (Braginsky et al., 2019; Gentner, 1982; Gillette et al., 1999; Goodman et al., 2008; Hansen, 2017; Hao et al., 2015; Naigles & Hoff-Ginsberg, 1998). Finally, early on, children are more likely to learn nouns and social words (e.g., games/routines, kinship terms), less likely to learn predicates (i.e., verbs and adjectives), and even less likely to learn closed-class words, such as articles (e.g., *the*) or prepositions (e.g., *from*) (Bornstein et al., 2004; Caselli et al., 1995; Fenson et al., 1994; Gentner et al., 2001; Sandhofer et al., 2000). This remains true even after controlling for differences in frequency and imageability (e.g., Hansen, 2017; McDonough et al., 2011).

There is, however, a curious gap in this literature. Taken at face value, most theories of word learning predict that frequency and imageability should interact, particularly early in development. To learn a word, you must both encounter it and be able to derive something useful from that encounter. For novice word learners, each instance of a word whose referent is easily observed or imagined is likely to be more learnable than each instance of an abstract word. Thus, we should expect to see an over-additive interaction between these variables. Encountering a word frequently is only useful if the child can infer something about its meaning, and even the most imageable word cannot be acquired if the child never encounters it. But to the best of our knowledge, this prediction has gone unnoticed in the literature, and no one has ever reported an interaction of this kind.

The present study has three goals. The first is to examine the interaction between frequency and imageability in early word learning. The second is to explore whether this interaction changes over developmental time. As children mature, they may be better able to learn words through fewer exposures, apprehend more abstract concepts, or use other information to acquire abstract words. Such changes could reduce the importance of words being both frequent and imageable resulting in a smaller interaction. Finally, we look at whether the syntactic category of a word continues to predict its acquisition once this interaction is also accounted for. Because words from different syntactic categories differ substantially in terms of their average frequency and imageability, it is conceivable that the category effects will disappear when we model the interaction as well. In the remainder of the introduction, we discuss: 1) why common theories of early word learning predict an over-additive interaction between frequency and imageability, particularly early in development; 2) why syntactic category may play a role independent of frequency or imageability; 3) the prior literature on which properties of words predict early acquisition; and 4) the goals of the present study.

### Contemporary Theories of Word Learning Predict a Frequency by Imageability Interaction

Theories of early word learning differ in many ways. Some theorists propose that concepts are constructed as words are acquired (e.g., Smith et al., 2002; Xu, 2002), while others propose that concepts are typically acquired prior to word learning (e.g., Gillette et al., 1999; Snedeker et al., 2012; Snedeker & Gleitman, 2004). In some theories, the process of word learning is gradual and continuous (e.g., Smith & Yu, 2008; Yu & Smith, 2007), while, in others, it is sudden and discrete (e.g., Medina et al., 2011; Trueswell et al., 2013). Nevertheless, every viable theory of word learning is the same in one respect: to acquire a new word, the learner must encounter the word form in contexts in which they can infer its meaning. This is necessary for the child to learn the form, the meaning, and their relation to one another.

This basic shared truth explains why frequency should affect word learning: children are more likely to encounter common words than uncommon ones, and thus, all other things being equal, they are more likely to encounter common words in interpretable contexts. In this simple model, imageability can be interpreted as a proxy for the degree to which the meaning of a word is likely to be accessible in any given situation. What makes more imageable meanings more accessible varies from theory to theory. In theories in which concepts are constructed as a part of word learning, imageability can be thought of as a proxy for the order in which concepts are learned (more concrete ones first, then more abstract ones). In theories in which concepts are largely constructed prior to word learning, imageability can be seen as a proxy for the ease with which the meaning of the word can be inferred from non-linguistic cues to the speaker’s intended meaning, such as eye-gaze, gesture, and the shared physical environment (Gillette et al., 1999; Snedeker et al., 2012).

Critically, this shared understanding of word learning—that it depends on encountering interpretable instances of the word in the input—makes two additional predictions. First, early in word learning, there should be a strong over-additive interaction of frequency and imageability such that the child primarily acquires words that are both frequent and imageable. A child might hear a word a million times, but if they cannot represent or identify its meaning, then they cannot map the meaning to the form. For example, *the* is the most frequent word in English (Davies, 2008), but it is unlikely that a child could determine its meaning until they have learned the meanings of many other English words. Conversely, a word can be conceptually accessible to the child and highly imageable, but if the child never encounters the word, they will not acquire it. For example, the concept of a *yurt* is concrete and presumably accessible to young children, given the relevant experience. But few English-speaking infants hear the word often enough to learn it. Thus, given the shared assumptions of contemporary word learning theories, early in life children should primarily learn words that are both frequent and imageable. More precisely, the average value of each learning instance should monotonically increase with its accessibility (by proxy, its imageability) resulting in an over-additive interaction.

Second, the strength of this interaction should decrease over time. The precise reason for this prediction varies across theories. In theories in which concepts develop in parallel with early words, more abstract concepts should gradually become accessible as the child gets older, allowing abstract but frequent words to be acquired. This would result in a larger frequency effect, a smaller imageability effect and a smaller interaction. On theories where vocabulary acquisition is largely a mapping problem, like syntactic bootstrapping, the imageability bottleneck disappears as children acquire new sources of information to solve the mapping problem (see e.g., Gillette et al., 1999; Gleitman & Gleitman, 1992; Snedeker & Gleitman, 2004; Snedeker et al., 2007). When children first begin decoding their language, they must infer a word’s meaning from social cues and extralinguistic context, which strongly favors more imageable words. Older children can use the co-occurrence of a novel word with known words and the syntax of the sentence to make inferences about the message that the speaker is conveying and the likely meaning of the unknown lexical items. This allows a wider range of contexts to be relevant for word learning, resulting in a smaller imageability effect, a persistent frequency effect, and a smaller interaction (since less imageable words can now also benefit from higher frequency).

### Syntactic Category Effects in Early Word Learning

One of the most robust findings in developmental psychology is that composition of children’s vocabulary changes over time (Bornstein et al., 2004; Caselli et al., 1995; Fenson et al., 1994; Gentner et al., 2001; Kauschke & Hofmeister, 2002; Nice, 1925; Papaeliou & Rescorla, 2011; Schults et al., 2012; for recent meta-analysis, see Braginsky et al., 2019). Children’s very first words are primarily “social words,” such as names for people (“Mommy”), games and routines (“peekaboo”, “bye bye”) and common sounds (“meow”) (Caselli et al., 1999). As their vocabularies grow, an increasing percentage of the words they know are concrete nouns, such as the names of common objects (“cup”) or animals (“kitty”). The proportion of nouns peaks around the time the child produces about 100 words. After this, children begin to learn more predicates, including both verbs and adjectives (Bates et al., 1994). When the child’s vocabulary reaches about 400 words, there is a systematic increase in the proportion of closed-class items. This category consists of a diverse set of words that mark grammatical functions, which in English includes articles, question words, modals and auxiliary verbs, and prepositions.

This pattern has been documented most extensively in studies using parent checklists, such as the MacArthur Bates CDI in American English and related instruments in other languages (Bates et al., 1994; Bornstein et al., 2004; Braginsky et al., 2019; Caselli et al., 1995, 1999), and the benchmarks above refer to the number of words that the child produces, as assessed by these instruments. But the pattern is not unique to these checklists. The same broad shifts have been observed in diary studies, direct assessments, and measures of spontaneous production (e.g., Goldin-Meadow et al., 1976; Greenfield, 1978; Nice, 1925, for reviews see Gentner, 1982). While there is some variability in early vocabulary acquisition across languages and cultures—Italian infants learn more words for family members (Caselli et al., 1995) and Chinese children acquire more verbs (Tardif et al., 1999)—the shifts in vocabulary composition are remarkably stable across a wide range of languages and learning contexts (see Braginsky et al., 2019).

What is less clear is *why* there are syntactic category effects. There are two broad possibilities, which are not mutually exclusive. The first is that differences between the categories can be explained by differences in the frequency or imageability of words in that category. Across languages, closed-class words are typically less imageable than predicates, which in turn are less imageable than nouns (see e.g., Gillette et al., 1999; Hansen, 2017; Ma et al., 2009). Thus, differences in imageability pattern with differences in acquisition probability in the expected direction. The relationship between frequency and syntactic category is more complex: the individual nouns that children encounter are on average less frequent than the individual verbs, which in turn are far less frequent than the closed-class items (Goodman et al., 2008; Ma et al., 2009; Sandhofer et al., 2000). In short, differences in frequency across these classes pattern in the opposite direction of what would be needed to explain the syntactic category effects. When both frequency and imageability are included in analyses both are reliable predictors, but they do not eliminate the syntactic category effects (see e.g., Hansen, 2017). However, as we noted earlier, there are strong theoretical reasons for expecting that these two variables are not in fact independent but instead have an over-additive interaction. Such an interaction could potentially account for the category effects: it would, on average, decrease the expected impact of frequency on closed-class words (which are highly abstract) and increase the impact of frequency on nouns.

The other possibility is that syntactic category effects reflect properties of the word learning process that cannot be reduced to frequency or imageability–features of acquisition that go beyond the simple consensus model described above. There are three lines of research that make specific predictions about syntactic category effects in early word learning.

First, syntactic category effects are predicted by theories in which early word learning is guided by strong biases to map words to particular kinds of meanings, since words belonging to favored categories should be acquired more readily than words with less favored meanings. For example, Markman’s whole-object constraint (1990), which leads children to assume that labels refer to whole objects, rather than to their constituent parts or properties, predicts that children should learn many nouns more readily than verbs (since object kinds are generally nouns). Related constraints like the shape bias, taxonomic constraint, and basic level bias, would facilitate noun learning as well (Golinkoff et al., 1995; Landau et al., 1988; Markman, 1990).

Second, syntactic category effects could arise from systematic differences in the position of words in utterances that might affect how easy it is to segment and represent word forms. For example, words that frequently occur in isolation are more likely to be learned before 15 months of age than words that rarely occur in isolation (Swingley & Humphrey, 2018). While words of all categories can occur in isolation, many social words are habitually used in this way (e.g., “hello”, “thanks” and “no”) and thus we might expect, all other things being equal, that very young children would be more likely to learn social words, than nouns, verbs, or closed-class items. For example, in English, common nouns often occur at the end of an utterance both because in transitive sentences objects occur after verbs and because new and focused information often appears in object position resulting in the use of nouns in this position rather than pronouns. Children generally process and remember utterance final words better than utterance medial words, presumably both because they are often longer and louder (making them easier to recognize) and because they are less likely to be affected by retroactive interference (Fernald et al., 2001; Peters, 1985; Slobin, 1985; Sundara, 2018).

Third, syntactic category effects are predicted on theories, like the syntactic bootstrapping hypothesis, in which words systematically vary in the nature of the evidence that we use to infer their meaning (Gillette et al., 1999; Gleitman & Gleitman, 1992; Landau & Gleitman, 1985; Snedeker & Gleitman, 2004). Syntactic bootstrapping is rooted in the observation that words from different syntactic categories tend to have different semantic functions which are reflected in their relationship to both the physical world and to the other words in an utterance. For example, nouns generally refer to specific individuals of the relevant kind. These kinds are defined by properties (e.g., *cat-ness*, *tree-ness*) which are independent of the event being described. Consequently, these meanings can often be inferred by mapping the word to the object without knowing anything about the rest of the sentence (Gillette et al., 1999; Snedeker & Gleitman, 2004; Medina et al., 2011). Predicates, in contrast, are used to describe relations between event participants, states that may change over time, or properties that are situationally relevant. Inferring the meanings of these predicates, even when they are concrete, often requires identifying the entities under discussion by using the nouns in the sentence and inferring the kind of event being described by using the syntactic structure of the clause (Gillette et al., 1999; Snedeker & Gleitman, 2004). The syntactic bootstrapping hypothesis argues that these systematic semantic distinctions result in a steady shift in the kinds of words that children are able to acquire as their knowledge of the language grows. While these differences in semantic function may be correlated with differences in imageability, there is no reason to think that ratings of this kind would fully capture these effects.

### Prior Findings on the Predictors of Early Lexical Acquisition

As we noted earlier, there is robust evidence that the imageability and frequency of a word predict how early it will be acquired. Imageability is typically measured by asking adults to rate the ease with which they can bring a mental image of the referent to mind (imageability) or the degree to which the word refers to things that can be experienced through the senses (concreteness, Paivio et al., 1968). These two kinds of ratings are very highly correlated (see Methods) and appear to tap into the same underlying psychological construct (e.g., Scott et al., 2019). For this reason, we will refer to the construct as *imageability*, and use the word *concreteness* only when describing individual studies using this specific variable. Imageability is a highly robust predictor of early word learning, producing strong effects regardless of whether other variables such as frequency or syntactic category are present in the model (Hao et al., 2015; Ma et al., 2009; McDonough et al., 2011; Smolík, 2019).

The effect of frequency is a little more complicated, but it emerges reliably so long as two conditions are met. First, because frequency is negatively correlated with imageability and systematically different across syntactic categories (Hansen, 2017; Hao et al., 2015; McDonough et al., 2011), the simple correlation between frequency and age of acquisition (or probability of acquisition) is often weak or even absent (see e.g., Goodman et al., 2008). When imageability or syntactic category are included in the analysis, or when the analysis is restricted to a single syntactic category, then frequency effects are consistently present and typically quite large (Braginsky et al., 2019; Goodman et al., 2008; Swingley & Humphrey, 2018). Second, word frequency distributions are highly skewed such that most words are low frequency, while a few are highly frequent. Psycholinguistic research has consistently found that the cognitive and linguistic effects of frequency are best captured on a log scale (Baayen, 2001). Studies in which frequency is not log transformed often fail to find frequency effects on the order of acquisition (Ma et al., 2009), while those with the transformation find large frequency effects (Swingley & Humphrey, 2018).

Several recent studies have simultaneously explored the effects of several different predictors on the probability that word will be acquired. Four papers are particularly relevant to the present project.

The first paper, by Braginsky et al. (2019), used a statistical approach similar to the present paper: models were constructed to predict the likelihood of a child producing (or understanding) a word, given the age of the child and a number of properties of the word itself. The authors constructed models for ten languages based on parent report data from the WordBank database, including English. For each language, they examined the subset of words that appear on both the Words and Gestures form for infants (8–16 months) and the Words and Sentences form for toddlers (16–30 months). The authors explored nine word-level predictors, as well as the interaction of each of these predictors with age. All predictors were entered into the model simultaneously. The strongest predictor, across languages and measures, was frequency. Concreteness and the interaction of age and concreteness also had robust effects on both comprehension and production. In addition, there were effects for measures of perceptual salience (e.g., isolated word frequency and utterance final frequency), phonological complexity (e.g., number of phonemes) and a rating of the degree to which the word is associated with babies. As their project differed in scope from our own, the authors did not test for the interaction between frequency and concreteness, or for effects of syntactic category independent of frequency and concreteness.

The second study, by Swingley and Humphrey (2018), used samples of an individual child’s input to model the probability of that particular child producing or understanding a given word by 12 or 15 months, as measured by the CDI Words and Gestures form. The authors conducted multilevel logistic regressions with eleven predictors including a three-way syntactic category distinction (parallel to the one above), frequency and concreteness. The analysis began with the full model and predictors were removed based on criteria that included significance and improved model fit. Only interactions with the syntactic category variable were tested. For comprehension, the authors found that word frequency was the strongest predictor, with concreteness, syntactic category and frequency in isolation also predicting acquisition. Word category influenced the size of the effect of frequency in isolation, which was found to be stronger for closed-class items than nouns. For production, frequency, frequency in isolation and syntactic category were reliable predictors. No effect of concreteness was observed. As in Braginsky et al. (2019), the interaction between frequency and imageability was not examined.

The third study, by Hansen (2017), tested a model predicting word learning in a sample of 6574 children used to norm the Norwegian CDI. This model included frequency, imageability, word length, word class, and all their pairwise interactions, for a total of 10 parameters. Nouns and predicates taken from the Norwegian CDI Words and Gestures (8–18 months) and Words and Sentences (16–30 months) were included in this model. In contrast with the previous studies, which used mixed models, the authors conducted an item-based analysis predicting the age (or vocabulary size) by which 50% of the children knew the word. All ten factors were simultaneously included in the model and the beta-values and significance of each factor was interpreted (as in the Braginsky et al., 2019 study). Frequency, imageability, word class and word length all predicted an earlier production of a word. Critically, there was no reliable interaction between frequency and imageability.

Finally, Smolík (2019) used frequency, imageability, word length, neighborhood density, and word class to predict the age of acquisition for nouns and predicates in Czech. Their study examined a sample of 493 children who were administered the Czech CDI Words and Sentences form. They fit a multiple regression model using all their predictors with age of acquisition as their dependent measure, similar to Hansen (2017). As predicted, they found negative relations between age of acquisition and frequency and between age of acquisition and imageability. Next, they fit models containing all their main effects plus interactions, including frequency-by-imageability. Like Hansen, they found no significant effect of this interaction in their model. The negative results reported by these two studies raise the possibility that this straightforward prediction of current theories of word learning might well be false.

### Current Study

The present study addresses the three questions mentioned previously.

First, we will investigate whether the interaction between frequency and imageability predicts word learning over a model that considers the two independently. Although most theories logically predict an interaction between these two factors, to the best of our knowledge the Hansen (2017) and Smolík (2019) papers are the only ones that have included this interaction term. The failure to find this interaction, despite the large data sets (n = 6574 in Hansen), raises the possibility that the two factors are truly independent, challenging our current theories of word learning. Alternatively, the absence of this interaction could reflect limitations of these studies such as: the omission of closed-class words and social routines from this analysis; the use of a summary variable (age by which median child acquires a word) rather than individual acquisition data; and the decision to test the interaction of imageability and frequency in the context of a single model with several other interaction terms, many of which are not reliable predictors and have no obvious theoretical motivation. The present study addresses these concerns by taking a theory-based hierarchical modeling approach to explore the effects of the three most robust and well-studied variables on the probability that an individual child will be reported to produce a word.

Second, we will explore whether the magnitude of this interaction changes with age. As children become more linguistically competent, we expect that they will be increasingly able to learn less imageable but more frequent words because their ability to infer the meaning of these abstract words will improve. We also might expect that words that are relatively low in frequency but high imageability are acquired as time goes by and children encounter these rare but often memorable concepts (e.g., pumpkins and snowsuits). Prior studies have not addressed this question either because they used dependent variables or data sets that do not allow for comparison across age groups (Hansen, 2017; Smolík, 2019; Swingley & Humphrey, 2018) or because they did not explore the interactions between word-level variables (Braginsky et al., 2019).

Finally, we will also determine whether there are differences between syntactic categories above and beyond the effect of imageability, frequency and their interaction. Prior studies have consistently found syntactic category effects when frequency and imageability are present in the model (see e.g., Hansen, 2017; Swingley & Humphrey, 2018). These effects, however, might disappear when a predictor is included (the interaction term) that can account for the early acquisition of words that are both highly frequent and imageable (e.g., very common nouns), without also predicting the precocious acquisition of function words and light verbs that are frequent but quite abstract.

We chose to focus solely on the production data from the Words and Sentences form of the CDI for two reasons. First, parent reports of children’s production have greater test-retest reliability and concurrent validity than parent reports of comprehension (Bates et al., 1988; Fenson et al., 1994), perhaps because they ask about a behavior that is directly observable rather than a cognitive state that must be inferred. Second, developmental shifts in lexical production are most readily observed in the period between 16 and 30 months during which combinatorial speech emerges, the proportion of nouns rises and declines, and the closed-class vocabulary begins to emerge. The words on the Words and Sentence forms were selected to capture the changes that happen across this age range. Many of these words are rarely reported in the speech of younger infants and thus do not appear on the Words and Gestures form for 8- to 16-month-olds. Thus, if we expanded our age range to include data from younger children, but focused solely on the items that appear on both forms, we would have decreased our ability to accurately assess the factors shaping word learning during these critical transitions.

## METHODS

### Participants

We analyzed 1461 children from the MacArthur Bates Communicative Development Inventory norming study (Fenson et al., 1994, 2007). Subjects were between the ages of 1;4 and 2;6 (*M* = 23.01, *SD* = 4.09) and consisted of 734 boys and 727 girls. Of these subjects, 697 were first born, 490 were second born, and 269 were later born (birth order information from 5 children was not available). All children were monolingual English speakers with no reports of atypical development. Maternal education ranged from 6–18 years (*M* = 14.32, *SD* = 2.36), with 989 having completed college and 471 having not (education information was not available for one of the mothers).

### Measures

#### Vocabulary.

Data was collected using the Words and Sentences form of the MacArthur Bates Communicative Development Inventory (CDI), a parent report of children’s expressive vocabulary between the ages of 16 and 30 months. The form includes a checklist of 680 lexical items organized into 22 semantic categories. For each item, parents indicate whether their child has spontaneously produced this word previously. Vocabulary was coded as a binary variable, 1 if the parent indicated that the child says the word, and 0 otherwise. Vocabulary size in this sample spanned the full range of the instrument (*M* = 279.5, *SD* = 198.2, range: 0–680). The data was obtained from the WordBank online database (Frank et al., 2017).

#### Spoken Frequency.

The spoken log-frequency of each vocabulary item was estimated from speech corpora of American English drawn from CHILDES (MacWhinney, 2000). Transcripts were included in the analysis if 1) they had the target child marked as *CHI, and 2) the age of the child in the transcript was available. The final analysis included 1049 transcripts of child-directed speech for children under 30 months (2,237,915 words), and 1067 transcripts for children over 30 months (2,607,223). Total word frequency, as well as CDI word frequency, was gathered using FREQ and FREQMERG programs in CLAN. Nine words from the CDI were omitted from the analysis because they were listed in a way that made it unclear how to calculate frequency in a consistent and unbiased manner. These words included three items from the People subsection that identified a variable that would require hand coding of corpora (e.g., “child’s own name”) and six items from the “Games and Routines” section that involved multi-word utterances that were likely to be produced in variable ways across families and contexts (e.g., “gonna get you”). Thus 671 words were retained in our final analysis.

In determining the frequency of each word, we included morphological variants belonging to the same syntactic category (e.g., *walk*, *walks*, and *walking*). A coder went through each of the words and determined whether they were plausibly polysemous in child-directed speech. Our enumeration of meanings was conservative: we required a distinction in syntactic category (noun vs verb) or ontological category (animal vs food). Of the 671 words, 135 were identified as potentially polysemous. For every word, we pulled 10 instances from CHILDES at random (each from a different transcript) and coded whether the word has the intended meaning. If all 10 instances of the word had the intended meaning, we calculated frequency based on FREQ with no modifications. If any of the instances had another meaning, we coded a total of 100 instances from CHILDES for their meaning and prorated the totals from the FREQ calculations based on the percentage that had the intended meaning.

#### Imageability.

To obtain judgments about word imageability, 30 adult participants were recruited and surveyed on Amazon’s Mechanical Turk. Imageability was defined as the ease with which a word arouses a mental image or sensory experience. For example, *apple* is a highly imageable word, while *fact* which does not easily produce a mental image is not (Paivio et al., 1968). Each participant was asked to rate the imageability of 340 CDI items, or half of the inventory, on a seven-point Likert scale (see Table 1). Each word was disambiguated for its syntactic category to ensure participants were rating the intended meaning.

**Table 1.** Summary word statistics by syntactic category: number of words, log frequency, imageability, and average percentage of known across participants.

	Total	Social	Nouns	Predicates	Closed
# of Words	671	57	334	166	114
Frequency (*SD*)	5.37 (1.85)	5.19 (1.9)	4.64 (1.46)	5.65 (1.66)	7.2 (1.73)
Imageability (*SD*)	4.89 (1.85)	4.72 (1.6)	6.32 (0.45)	4.03 (1.17)	2.03 (1.07)
Proportion known	0.41	0.54	0.46	0.37	0.25

#### Concreteness.

Using the same procedure we used to collect imageability, we also collected judgements about concreteness. Concreteness was defined as the ease with which a word can be felt, heard, touched, or otherwise perceived in the world (Paivio et al., 1968). For example, *cake* can be perceived across many different senses (e.g., tasted, seen, smelled), whereas *think* is more difficult to describe in sensory terms. As with imageability, each participant was asked to rate the concreteness of half of our CDI inventory on a seven-point Likert scale. We found that concreteness and imageability were found to be almost perfectly correlated with one another (*r*(669) = 0.99, *p* < 0.001). Because of this, only imageability judgements were used in our analyses.

#### Syntactic Category.

Following previous studies, words were classified as either nouns, predicates, social words, or closed-class items based on which semantic category they fell into according to the CDI (Bates et al., 1994; Caselli et al., 1995). We deviated from the classification schema of these studies by including two previously excluded categories, Time Words and Places to Go, in our analysis as closed-class items and nouns respectively. This was done to increase the coverage of our analyses. The words in each of these sections largely fall within these syntactic categories and appear to develop along the same time course (Snedeker et al., 2007, 2012).

Social words included Sound Effects, Games and Routines, and People. Some of the words in this category, such as names for people, are clearly nouns in the adult lexicon. We chose to code them as social words for three reasons: 1) this was the coding scheme used in the prior Bates paper on which we based our analyses (see e.g., Bates et al., 1991); 2) Based on this coding scheme Bates and colleagues had proposed that Social Words were typically produced earlier than nouns, and it was this observation that we hoped to explore further in our analyses; 3) Many of these words are also initially used by children as proper names rather than common nouns (“Mommy” vs. “a mommy”) and thus may not require the same degree of generalization as other nouns.

Nouns included Animals, Vehicles, Toys, Food and Drink, Clothing, Body Parts, Small Household Items, Furniture, Outside Things, and Places to Go. Predicates consisted of Action and Descriptive Words, corresponding to verbs and adjectives respectively. The remaining categories (Time Words, Pronouns, Question Words, Prepositions, Quantifiers, Helping Verbs, and Connecting Verbs) were included together as closed-class items. Categories were coded as dummy variables (1 = membership, 0 = non-membership). In Figure S1, we have plotted the compositions of children’s vocabularies by syntactic categories as their vocabularies grow.

### Statistical Analysis

Mixed effects logistic regression models were fitted to the data using *glmer* in the R package *lme4* (Bates et al., 2015; R Core Team, 2017), with whether a word was known or not known by a given child serving as the response variable. We used Nelder-Mead optimization to fit our regression models (Nelder & Mead, 1965). We found that our models failed to converge when using the default optimization method that *lme4* runs. To ensure our results were not affected by convergence failure, we used the function *allFit* to compare our regression coefficients across different optimization methods. We found that each method produced almost equal fixed effects estimates (see Figure S3 in Supplementary Materials). The base model contained imageability, log-frequency, and child age in months, and random intercepts by subject and by item. Next, we introduced the imageability-frequency interaction term, and finally syntactic category. The best-fitting model was determined by conducting a forward stepwise model comparison using log-likelihood ratio testing as each predictor was added. For models containing category covariates, closed-class items were used as the contrast case. In addition to these main models, we also ran an analysis exploring how age interacts with our other predictors. This analysis was performed to investigate whether the effect of frequency-by-imageability changed over development. Starting from the original model containing our frequency-by-imageability interaction term, we introduced each by-age interaction, going from imageability-by-age to frequency-by-age, and finally to the three-way interaction term, comparing each model in a stepwise fashion as we had in our original analysis. The models that we report here include random intercepts for both participants and items. For each of our models, all continuous variables were centered and standardized, and we converted the resulting logistic regression coefficients to odds ratios for interpretability.

### Supplementary Analyses

We also conducted another analysis where we divided our sample into three age groups (16–20 months, 21–25 months, and 26–30 months) and conducted our primary analysis on each of these datasets separately (rather than introducing age as an interaction term). The results of these analyses can be found in Tables S1, S2, and S3.

## RESULTS

### Summary Statistics

**Figure 1.**
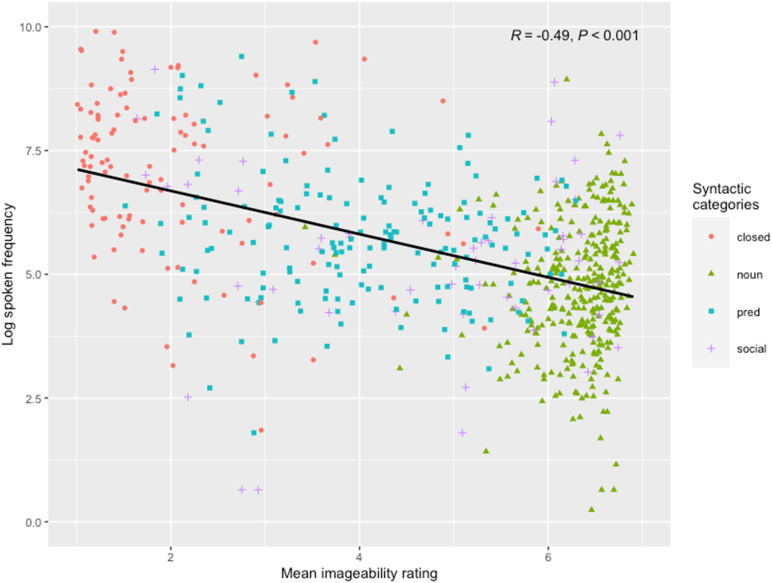
Words plotted against imageability and log frequency by syntactic category.

Mean values for imageability, frequency, and word knowledge by syntactic category can be found in Table 1 (see Figure S2 in Supplementary Materials for the distribution of imageability and frequency values by syntactic category). Word imageability and log frequency were found to be negatively correlated (Figure 1).

### Is There an Interaction Between Frequency and Imageability?

To address our first research question, we constructed a series of models of gradually increasing complexity (see Table 2). We began with our two-factor model containing both frequency and imageability (Model 1). We found that both frequency (*β* = .85, *SE* = .07, *OR* = 2.34; *p* < .001) and imageability (*β* = 1.07, *SE* = .07, *OR* = 2.91; *p* < .001) were significant predictors of word knowledge in this model. As in previous studies, we found that imageability and frequency are negatively related (*r*(669) = −.518, *p* < .001), which explains why the odds ratio for both frequency and imageability increases when both are included in the model.

**Table 2.** Stepwise Mixed Effects Logistic Regression Models Predicting Children’s Word Knowledge.

	Model 1	Model 2	Model 3
Intercept (Std. Err)	−0.82*** (0.08)	−0.64*** (0.08)	−2.72*** (0.17)
Age	1.65*** (0.05)	1.65*** (0.05)	1.65*** (0.05)
Imageability	1.07*** (0.07)	0.93*** (0.07)	0.15 (0.09)
Frequency	0.85*** (0.07)	0.92*** (0.06)	1.07*** (0.06)
Frequency × Imageability		0.37*** (0.06)	0.31*** (0.05)
Predicate			1.49*** (0.17)
Noun			2.81*** (0.22)
Social			3.32*** (0.22)

Improved Fit?		Yes	Yes
ANOVA *χ*^2^		42.45***	144.49***

Best fit at each step bolded. Best fit overall boxed.

′ *p* < 0.1, * *p* < 0.05, ** *p* < 0.01, *** *p* < 0.001.

**Figure 2.**
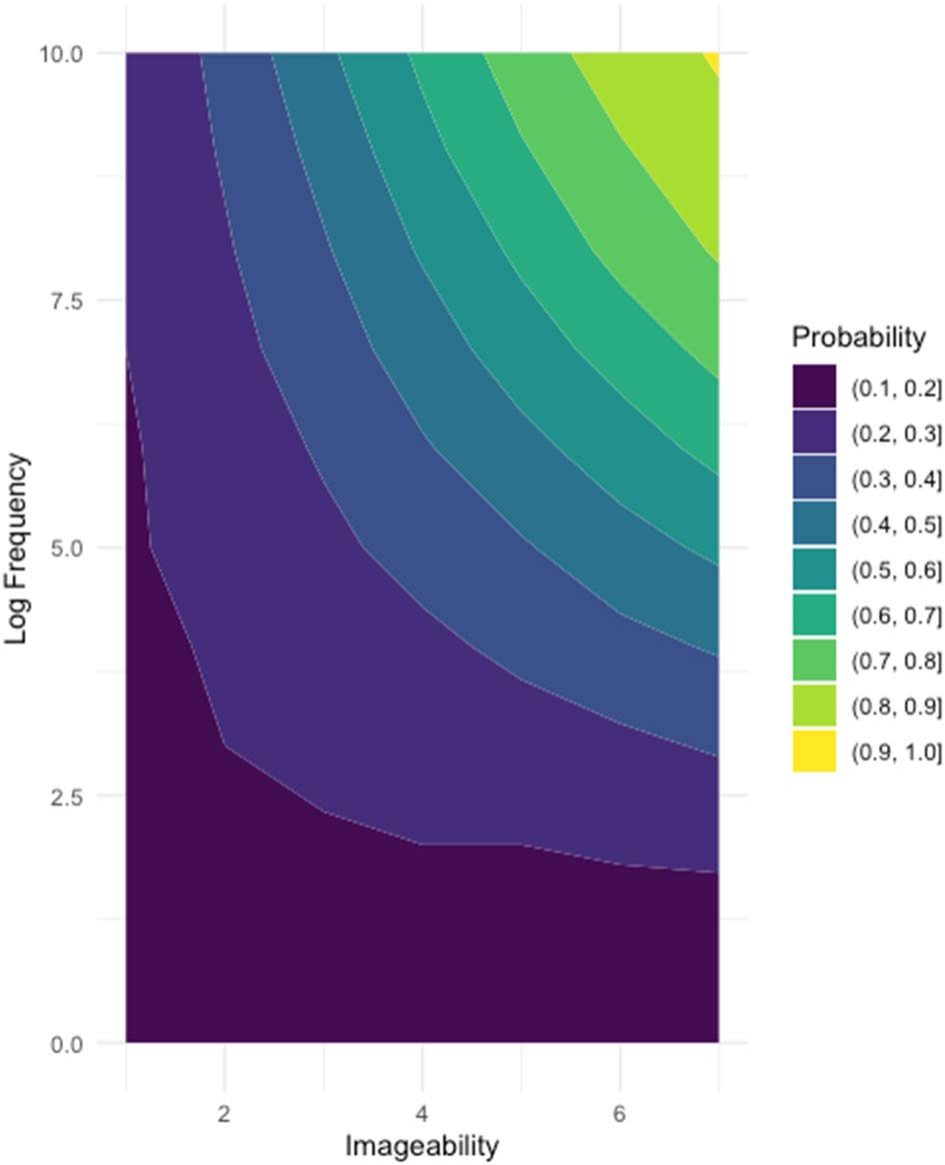
Contour plot illustrating the probability words are known given their imageability and log frequency for our interaction model.

We then added the frequency by imageability interaction term to our two-factor model (resulting in Model 2). This model was a significant improvement over the additive model (*χ*^2^(1) = 42.45, *p* < .001), and the interaction term was significant and positively predictive of word knowledge (*β* = .37, *SE* = .06, *OR* = 1.44; *p* < .001). The positive beta value indicates that this interaction is over-additive: the probability of learning a word is greater when it is both frequent and imageable. In Figure 2, we can see over-additive effects of the imageability-by-frequency interaction on the probability a given word is known.

### Do the Differences Between Syntactic Categories Persist After Controlling for the Interaction of Frequency and Imageability?

Next, we added a final model to this series that examined the effects of syntactic category in word knowledge (Model 3), by introducing the three category variables (using closed-class items as our contrast case). The addition of these variables improved fit relative to the interactive model (*χ*^2^(3) = 144.49, *p* < .001), demonstrating that the differences in acquisition between different syntactic categories cannot be attributed solely to their differences in frequency or imageability. In this model, each of the three parameters for the contrasting syntactic categories was a significant predictor, indicating that social words, nouns, and predicates are each more likely to be acquired than closed-class items. The frequency-by-imageability interaction remained significant (*β* = .31, *SE* = .05; *OR* = 1.36; *p* < .001), suggesting independent contributions of this interaction and syntactic category to word learning.

Based on the prior literature we would also expect that nouns and social words would be easier to acquire than predicates and that social words would be easier to acquire than nouns. To test this possibility, we ran two more syntactic models (closely parallel to Model 3) on subsets of the data. The first, excluded all closed-class items and treated predicates as the contrast case with two syntactic category variables picking out nouns and social words. We then compared the fit to a version of our interaction model (parallel to Model 2) run on our dataset excluding closed-class items. We found that the addition of syntactic category as a predictor still improved our model fit after excluding closed-class items. Both noun and social word membership were positively predictive of word learning. We then replicated this analysis excluding both closed-class items and predicates and using nouns as our contrast case. Again, we found that the model with a syntactic category variable provided a better fit, demonstrating that children were more likely to know the social words than the nouns. Thus, we conclude that differences in the acquisition trajectories for these four categories cannot be attributed to differences in frequency and imageability, nor the interaction of the two variables.

### Does the Strength of the Frequency by Imageability Interaction Change Between 16 and 30 Months?

We also wished to examine whether age influences the impact of these predictors on word learning. To accomplish this, we began from our frequency-by-imageability model and added each by-age interaction term in a stepwise fashion (see Table 3). We found that adding imageability-by-age to our base interactive model (Model 3) improved model fit (*χ*^2^(1) = 52.61, *p* < .001). The effect of imageability on word-learning was slightly decreased in older children (*β* = −.03, *SE* = .004; *OR* = .97; *p* < .001). We also found that adding frequency-by-age to a model that already contained imageability-by-age (Model 4) improved model fit (*χ*^2^(1) = 207.79, *p* < .001). The effect of frequency on word-learning was increased in older children (*β* = .06, *SE* = .004; *OR* = 1.06; *p* < .001), while imageability-by-age became non-significant. To ensure the order in which we introduced these variables did not impact our results, we checked to see if frequency-by-age alone improved fit compared to Model 2. We found that adding this interaction term alone also improved model fit. Finally, adding our three-way interaction term, imageability-by-frequency-by-age to the model with both two-way age interactions (Model 5) also improved model fit (*χ*^2^(1) = 70.16, *p* < .001). In this final model, we found a *negative* three-way interaction of frequency, imageability, and age (*β* = −.03, *SE* = .004; *OR* = 0.99; *p* < .001), suggesting that the positive effect of the frequency-by-imageability interaction decreases with age.

**Table 3.** Stepwise Mixed Effects Logistic Regression Models Predicting Children’s Word Knowledge with Age Interaction (beginning with Model 2 from Table 1).

	Model 2	Model 3	Model 4	Model 5
Intercept (Std. Err)	−0.64*** (0.08)	−0.64*** (0.08)	−0.63*** (0.08)	−0.63*** (0.08)
Age	1.65*** (0.05)	1.65*** (0.05)	1.65*** (0.05)	1.64*** (0.05)
Imageability	0.93*** (0.07)	0.94*** (0.07)	0.93*** (0.07)	0.93*** (0.07)
Frequency	0.92*** (0.06)	0.92*** (0.07)	0.91*** (0.06)	0.91*** (0.06)
Frequency × Imageability	0.37*** (0.06)	0.37*** (0.06)	0.38*** (0.06)	0.38*** (0.06)
Imageability × Age		−0.03*** (0.004)	0.01 (0.005)	0.06*** (0.004)
Frequency × Age			0.06*** (0.004)	0.02*** (0.005)
Frequency × Imageability × Age				−0.03*** (0.004)

Improved Fit?		Yes	Yes	Yes
ANOVA ChiSq		52.61***	207.79***	70.16***

Best fit at each step bolded. Best fit overall boxed.

′ *p* < 0.1, * *p* < 0.05, ** *p* < 0.01, *** *p* < 0.001.

## DISCUSSION

The current study has three primary findings. First, we discovered that young children mostly learn words that are both frequent and imageable (or concrete). Specifically, the probability that a toddler will be reported to produce a word is predicted by the interaction between its frequency in child-directed speech and its imageability.

Second, we found that the magnitude of these effects changed with age. While the youngest children primarily learn words that are both imageable and frequent (e.g., *mommy*), older children benefit independently from each factor, acquiring words that are concrete but rare (e.g., *pumpkin*) or common but more abstract (e.g., *nice*). As a result, there was a reliable negative three-way interaction of age, frequency and imageability (indicating that this effect declined with age).

Finally, we found that a word’s syntactic category affected the likelihood that it was learned, even after controlling for imageability, frequency, and their interaction. Across many languages, it has been found that children tend to learn social words and nouns first, acquire predicates such as verbs and adjectives somewhat later, and learn substantial numbers of closed-class items even later (e.g., prepositions, articles, etc.). Our findings indicate that these differences are not merely a manifestation of the interaction between imageability and frequency.

As we noted in the Introduction, our first finding is predicted by all contemporary theories of word learning and thus this result does not favor one of these theories over the others. If experiences with imageable words are more informative than experiences with less imageable words, then we should expect the effects of frequency to be greater for the imageable words, resulting in an over-additive interaction. This is true on a theory in which infants incrementally refine statistical distributions over possible meanings (e.g., Smith & Yu, 2008; Yu & Smith, 2007), and it is true on a theory in which learning is driven by the subset of cases in which the context strongly favors a single meaning (Medina et al., 2011; Trueswell et al., 2013). Our discovery is, nevertheless, theoretically constraining. This expected interaction was a critical unnoticed and untested prediction of our theories of infant word learning. If it had turned out to be false, we would have been forced to re-evaluate these theories to make sense of how frequency could promote word learning to the same degree regardless of whether a word is concrete or abstract. For example, this might have led us to reconceptualize imageability as an attentional filter that could be overcome with greater frequency, with decrements in imageability trading off linearly with increments in log frequency.

In the remainder of this Discussion, we explore four issues raised by these findings, namely: 1) What do imageability ratings measure and how can we go beyond these ratings to understand the underlying cognitive processes that link this construct to word learning? 2) To what extent are these findings consistent with prior findings on the predictors of early vocabulary acquisition and what might explain the differences? 3) What are the plausible explanations for the observed effects of syntactic category, and how might they be tested? 4) What are the limitations of the current study and how might they be addressed?

### What is Imageability?

Imageability and its close cousin concreteness have a long history as variables in psycholinguistic studies stretching back to the early 1960’s (see Paivio et al., 1968 for review). Imageability is typically measured by asking people how readily a given word produces a mental image. Developmental theories, however, do not assume that image generation itself is the mechanism driving the correlation between imageability and word learning. Instead, it is treated as a proxy for one (or both) of two things. First, imageability can be viewed as an indirect measure of the degree to which a concept is accessible to a young infant. For example, infants might acquire the concepts encoded in highly imageable words prior to word learning, making these words easier to learn. In contrast, less imageable words might have meanings that the child can only conceptualize at a later stage of development making these words impossible to learn at earlier ages. Second, imageability could be a proxy for the informativity of each learning instance. Specifically, more imageable words are likely to have more consistent and memorable perceptual correlates, making it easier for the learner to figure out the circumstances that are reliably associated with word use, and thus the meaning of the word.

The present findings are compatible with either of these hypotheses. If imageability is a proxy for an absolute conceptual constraint, then we should expect to see an over additive interaction with frequency, because at any given age, frequency will only be beneficial for words that are conceptually accessible (high imageability words). This interaction should get smaller over time as more concepts become cognitively accessible, leading to an increase in the main effect of frequency. If imageability is a proxy for the average informativity of each instance, then an interaction is predicted because the incremental effect of each instance the infant encounters is greater for the more imageable words. As children learn more about their language and are able to use linguistic context to infer meaning, the relationship between imageability and informativity should break down, resulting in a smaller interaction and a larger effect of frequency.

While the present data is consistent with either hypothesis, we believe that there are independent reasons to think that the effect of imageability is primarily linked to informativity and not conceptual accessibility. Specifically, the early vocabularies of internationally adopted children, who begin acquiring English at 2;6 to 5;0, are quite similar to those of infants who are learning English as a first language (Snedeker et al., 2007, 2012). Since these older children presumably have access to the concepts available to other preschoolers, these findings suggest that primary driver of vocabulary composition is changes in informativity as one acquires a language, rather than maturational changes in the conceptual repertoire. Systematic testing of this hypothesis would require modeling the effects of imageability, frequency and their interaction in populations (like internationally adopted children) where age and linguistic knowledge are not confounded.

Building a cognitive model of word learning will ultimately require that we move beyond proxy variables like imageability and build working models of the conceptual substrate of early word learning and how infants link the speech acts they observe to conceptual representations. Until we have such models, these proxy variables may be useful, both for describing data patterns and generating hypotheses. But it would be a mistake to treat them as causal factors in our theory of development.

Recognizing the limits of human ratings as variables is helpful in thinking through the relationship between concreteness and imageability. In our norming study, we found that imageability and concreteness were almost perfectly correlated (see Scott et al., 2019). This tight correlation is somewhat surprising: concreteness is generally defined as a measure of how abstract a word is (a construct similar to conceptual accessibility) while imageability is defined as the ease with which it brings an image to mind (a construct that seems more intuitively linked to the informativity of contexts). The fact that the ratings are so similar could reflect a systematic overlap between two distinct and causally relevant variables or it could indicate that participants in our ratings tasks map these two instructions onto a single set of intuitions. Critically, whatever the explanation might be, our findings show that there is no reason to debate the relative value of these measures, and no support for the claims that they have a different underlying distribution (contra Kousta et al., 2011). When concreteness and imageability are measured through participant ratings, they are not meaningfully different.

### Integrating Our Study With Prior Work on the Predictors of Vocabulary Composition

Our study contributes to a growing literature on the role of imageability and frequency in early word learning. These studies fall into two categories: 1) studies that do not test for the interaction of frequency and concreteness but use a logistic analysis, like ours, to assess the effects of each predictor on the probability of acquiring the words. Our work complements this research and is broadly consistent with it (i.e., Braginsky et al., 2019; Swingley & Humphrey, 2018); and 2) studies that test for an interaction of frequency and concreteness on a summary variable (mean age of acquisition) and fail to find it (i.e., Hansen, 2017; Smolík, 2019). These studies reach conclusions that are very different from ours. We suspect that this reflects limitations of their summary variable.

#### Prior Studies of Frequency and Imageability Interactions.

Our findings and conclusions are in direct opposition to the findings of Hansen (2017) and Smolík (2019). Both papers considered the impact of frequency and imageability on word learning in a single language (Norwegian and Czech, respectively). Both papers used the age by which 50% of children were reported to produce a word as their dependent variable. Both authors found that although there are effects of both frequency and imageability on vocabulary, there is no evidence for an interaction between these factors.

We see three possible explanations for the difference between our findings and theirs. First, the difference in findings could, logically, be attributable to differences in how children acquire English, as opposed to Czech and Norwegian. Perhaps there are differences in the syntax of these languages, the properties of words, or in the kinds of input children receive in these communities that lead to a difference in acquisition outcomes. While this is a logical possibility, we think it is unlikely. We know of no property that Czech and Norwegian have in common that sets them apart from English. By the most obvious metrics, Norwegian and English are more similar to each other than either is to Czech (e.g., both are Germanic languages with relatively strict word order in which case is often unmarked). Furthermore, the main effects of frequency and imageability seem to be very similar across a wide range of languages (see Braginsky et al., 2019). Nothing in the Hansen or Smolík data suggests that these factors behave differently in Czech or Norwegian, so it would be surprising if their interaction was different. Finally, as we noted earlier, the interaction of frequency and imageability is a straightforward prediction of our shared model of word learning. If each instance of an imageable word is less informative, we should expect an over additive interaction in early word learning across linguistic environments. Therefore, we believe that linguistic differences cannot explain these conflicting results.

The second possibility is that the differences in the findings reflect differences in the words that were included in the analysis. In our study, we included nearly all words from the toddler CDI: social words (including animal sounds and interjections), nouns, predicates, and closed-class items. Both Hansen and Smolík included focused solely on nouns and predicates. Closed-class items are characterized by very high frequency and very low imageability (see Table 1 and Figure 1). Thus, they provide the data that is most challenging for a simple additive model: any model that includes a positive effect of frequency and no interaction term is likely to predict precocious acquisition of these terms. Social words, in contrast, vary considerably in their interaction term and in their mean age of acquisition. Excluding both classes of words could reduce the magnitude of the interaction effect.

Finally, the absence of an interaction term could reflect Hansen’s and Smolík’s decisions to use a constructed variable (median age of acquisition) and a linear analysis. The presence or absence of an interaction depends on the scale used to measure the dependent variable (as well as the scaling of the predictor variables). Our dependent variable was closely linked to the underlying structure of the data set (children are either reported to know a word or not) and the psychological process that we believe it represents (does the child have a stable mapping for the word that allows them to produce it). In contrast, the median age of acquisition provides a concise summary of the data, but it has no clear interpretation at a cognitive or behavioral level. Critically, these two dependent variables cannot be directly mapped to one another: a word could have a median age of acquisition of 24 months and be known by 40% of 18-month-olds, or it could be known by no 18-month-olds at all. Future work could explore these three possibilities by comparing logistic analyses with age of acquisition analyses within each language along with the effects of including or excluding social words and closed-class items from the analysis.

### Examining Syntax and Its Relation to Word Learning

Our findings shed further light on the consistent finding that children learn words from some categories before others. Specifically, our models demonstrate that these differences cannot be fully accounted for by the differences in imageability and frequency between the words in these categories or, critically, by differences in the interaction between imageability and frequency. Our findings on this point diverge from two other studies. Hansen (2017) found that syntactic categories did not improve prediction of median age of acquisition beyond imageability for Norwegian nouns and verbs. McDonough and colleagues’ (2009) found that the effect of syntactic category in English flipped direction when imageability was included in a model predicting median age of acquisition. This raises the question of why words in some categories are acquired before others. There are three broad classes of hypotheses that might account for this pattern.

First, the differences could reflect language specific learning biases that privilege some syntactic categories over others. For example, many of the word learning biases put forward by Markman and others–such as the whole-object bias, the taxonomic constraint, and the shape bias–would privilege hypotheses that would facilitate learning nouns for basic-level object and animal categories (Landau et al., 1988; Markman, 1990; Smith et al., 2002). For example, a child who initially assumes that a novel word picks out a taxonomic category of objects with a consistent shape, should close in on the right hypothesis when hearing “ball” but not when hearing “catch!”. To the extent that these biases are the product of early word learning (Smith et al., 2002), their effects should overlap with other factors (like imageability and frequency) that allow those first words to be acquired. Once the bias has been acquired, however, it could have effects on vocabulary composition that go beyond these variables (e.g., benefitting a noun more than a verb that is matched for frequency and imageability). This explanation seems most plausible as an explanation for why nouns are acquired before verbs and closed-class items.

Second, the category differences could reflect differences in the perceptual salience or position of words which make it easier for learners to segment them from the speech stream and learn their forms. Many social words are performatives that are likely to appear in isolation, a factor that is known to increase the probability of early acquisition (Swingley & Humphrey, 2018). Closed-class words tend to be short with reduced vowels. Closed-class words are also often in the same prosodic phrase as an adjacent content word, potentially making them more difficult to segment and perceive. In English, lexical nouns frequently appear at the end of the utterance (in object position) where they are longer and followed by a pause. Previous studies have found that length, position and use isolation are predictors of early learning (Braginsky et al., 2019; Swingley & Humphrey, 2018). Nevertheless, in models where perceptual factors and syntactic category are both included as predictors, the difference between nouns and verbs persists (Swingley & Humphrey, 2018). This suggests that syntactic category differences are not solely due to position. Additional evidence comes from studies of vocabulary acquisition in languages with verb final word order–like Korean and Japanese. Children learning such languages also acquire nouns, on average, earlier than verbs, suggesting that the difference between these two classes is not solely attributable to order (Bornstein et al., 2004; Frank et al., 2021; Kim et al., 2000; Ogura et al., 2006). Future studies should explore whether the effects of syntactic category, independent of position, persist when the interaction between imageability and frequency are included in the model: both in English and in languages with more verb-friendly syntax.

Finally, the effects of syntactic category on learnability could be linked to semantic differences between these classes which are not fully captured by measures like imageability. One candidate is the semantic function of different words. Most nouns in child-directed speech refer to entities (e.g., objects or animals) that are stable over time and space. Verbs, in contrast, refer to events which are short lived. The meaning of a noun is largely independent of the other words in the sentence (e.g., a piano is a piano regardless of whether it is played or sat on), while the meaning of a verb often depends on the arguments it takes (e.g., playing a piano vs playing a game). Most nouns in children’s early vocabulary group individuals into categories (i.e., kinds) on the basis of stable properties (e.g., cats generally continue to be cats and cups continue being cups) while verbs might apply to an individual one moment but not the next (e.g., in a good conversation the listeners and the talkers are constantly changing). For these reasons, it is difficult for even knowledgeable adults to infer the verb that is being used in a sentence from visual context alone (Gillette et al., 1999; Snedeker & Gleitman, 2004). In contrast, many of the nouns in child-directed speech can be easily identified just from the visual context (Gillette et al., 1999; Medina et al., 2011). Verbs become far easier to identify, when adults are given information about either the syntax of the sentence, or about the nouns that occur with the verb (Gillette et al., 1999; Snedeker & Gleitman, 2004). This suggests that knowing some nouns may be a prerequisite to learning many verbs. Difference in meaning, reference and semantic complexity could also play a role in precocious acquisition of social words and the delayed acquisition of closed-class items. Many social words are performatives (choo choo, bye). These words do not necessarily have a referent (or a syntactic category). They are steps in a ritual, to be uttered at the right time and in the correct context. Thus, a child can learn them without identifying the adult conceptualization of the event, breaking it into pieces and mapping the word to the correct conceptual chunk. Instead, the child just has to identify the contexts in which the ritual happens and when they should perform their part. Closed-class items, in contrast, are often modeled as high-level functions that take nouns, verbs or even whole clauses as their arguments. While these semantic dimensions are clearly correlated with imageability, this correlation is unlikely to be perfect (“hi” is abstract but not semantically complex). Future work could explore the role of factors that capture these core conceptual distinctions and their relation to imageability, syntactic category and the acquisition trajectory.

### Limitations

There are several limitations to this study. First, we only explored vocabulary development between the age of 16 and 30 months. While our theories of word learning predict that this interaction should be present in even younger children, we chose not to include the norming data for the infant CDI (ages 8–16 months) which assesses both comprehension and production. This decision reflected three considerations. First, many children are reported to produce few or no words at these ages, limiting the power of a model focused solely on production in this age group. Second, while there is evidence validating the CDI comprehension measure as an aggregate variable, there are also reasons to believe that it is a less accurate measure at the level of individual words. When parents are asked what words their child says, they may search their memories for what they have heard. When they are asked what words their child understands, they must make more complex inferences based on children’s responses to whole utterances in rich contexts. In the absence of clear data, a parent may rely on their implicit theory of development (or their child) rather than concrete experiences. Finally, and most critically, the infant CDI may not include the range of words needed to observe this interaction. The infant form contains a subset of the words from the toddler CDI. Many of the words that are absent from the infant CDI are words that are extremely high in frequency, extremely low in imageability, and rare in early speech (e.g., *the*, *a*, *she*, *he*, *think*). Others are words that are low frequency but imageable (*pumpkin* and *popsicle*). We suspect that this reflects that procedure by which the instruments were constructed: words were initially selected based on their appearance in records of children’s early vocabularies and were retained as the instrument was refined based on their ability to capture individual and developmental change. As a result, words that were rarely uttered by children under 16 months were eliminated from the infant form. Our findings, based on the youngest kids tested with the toddler form, suggest that at even earlier ages, children are primarily producing words that are both imageable and frequent. But this pattern would be difficult to detect in an instrument in which few of the other words remain. Nevertheless, our current theories of word learning clearly predict that this interaction should be present in the youngest word learners and in comprehension as well as production, provided that we have valid measures that include a wide-enough range of the words. The concerns raised above suggest that future studies should also consider analyzing children’s vocabularies relative to all words that they have been exposed to, rather than relative to a set of words that other children acquired fairly early in life. Doing so would require different methods of data collection.

A further limitation of our study is that it focuses solely on English. Braginsky and colleagues’ analyses of CDI data from 10 languages strongly suggests that predictors of acquisition are broadly similar across languages. There are two reasons, however, to question whether the patterns that we observed here would appear in other languages. First, as we noted earlier, the absence of a frequency-by-imageability interaction in Hansen (2017) (Norwegian) and in Smolík (2019) (Czech) raises the possibility that this effect varies across languages or learning environments. Second, there is a large literature exploring the degree to which there are cross-linguistic differences in syntactic category acquisition. For example, the early bias for nouns does not seem as pronounced in Mandarin Chinese as in other languages (Braginsky et al., 2019; Hao et al., 2015). This phenomenon has been tied to different properties of the language. For example, predicates seem to receive higher imageability ratings on average in Mandarin than in English, perhaps making these words more easily learned at an earlier age (Ma et al., 2009). In addition, caregivers speaking these languages seem to produce more predicates in child-directed speech, making them more accessible earlier on (Tardif et al., 1999). Mandarin also permits subject and object omission, meaning that predicates are more likely to be encountered alone (Lee & Naigles, 2005). An analysis exploring syntactic category effects in relation to imageability, frequency, and their interaction, could help address the question of whether there are differences in the word learning strategies of the Mandarin and English learners, or just differences in the outcomes due to differences in the inputs.

Finally, our analyses did not incorporate many of the variables that have been explored in previous papers. As a result, we cannot assess the degree to which the interaction that we observed can account for these effects or whether controlling for these variables would eliminate the syntactic category effects. The value of such analyses depends on the following: the degree to which we believe that the variable in question is available to young children, whether we have a model of how it might influence acquisition (allowing us to scale the variable appropriately and focus on the most relevant interactions), and our understanding of how the effect of that variable might change over development. From this perspective the variables that seem most critical for future models are phonemic length and complexity and measures of perceptual salience such as frequency in isolation and frequency in utterance final position. Complexity might be construed as a filter on production, affecting that probability that the child who has successfully mapped a word (perhaps on the basis of its frequency and imageability) is able to produce a close enough approximation to be credited with using it. This could result in a three-way interaction. Curiously, neither Braginsky et al. (2019) nor Swingley and Humphrey (2018) found that measures of complexity (length and phonotactic probability, respectively) were reliable predictors of English language production. However, Braginsky found length to be a robust predictor of production across languages. This could potentially reflect differences in cultural practices around the interpretation of child utterances. Measures of perceptual salience, in contrast, have been consistently associated with higher probability of early acquisition (Braginsky et al., 2019; Swingley & Humphrey, 2018). These are most readily construed as modulators of frequency or alternative measures of frequency: variables that affect whether a given instance of a word is perceived at all at a given age. For this reason, we would expect that they would interact with imageability and age.

### Conclusion

Understanding the course of early vocabulary acquisition is central to refining our theories of language development and conceptual development. Previous work had found that children’s early word learning is affected by both frequency and imageability (or concreteness). The present study finds that this description is incomplete: early vocabulary is shaped by the interaction of these variables such that young children are primarily learning words that are both highly common and highly imageable. While this interaction was a straightforward prediction of contemporary word learning theories, there was no prior evidence supporting it. In addition, we find that this interaction declines with age and does not fully account for the acquisition differences across syntactic categories.

## ACKNOWLEDGMENTS

This work unfolded over many years. We would like to thank: the audiences at BUCLD and ISIS (now ICIS) for their questions and encouragement; Daniel Swingley and Felipe Smolik for more persistent interest; Michael Frank and Mika Braginsky for their advice on constructing our statistical models; and Harvard University Talley Fund for financial support of the research. This research was supported by a grant from the National Science Foundation (BCS-0418423).

## AUTHOR CONTRIBUTIONS

J.R.C.: Conceptualization: Equal; Formal analysis: Lead; Investigation: Supporting; Methodology: Equal; Writing: Lead. M.Z.: Conceptualization: Supporting; Investigation: Supporting; Methodology: Supporting. J.C.: Conceptualization: Supporting; Investigation: Supporting; Methodology: Supporting. J.S.: Conceptualization: Equal; Formal analysis: Supporting; Funding acquisition: Lead; Investigation: Lead; Methodology: Equal; Writing: Supporting.

## DATA AVAILABILITY STATEMENT

Our data is fully available via OSF: OSF | Imageability and Frequency of Words in Children’s Early Vocabulary.

## Supplementary Material



## References

[bib1] Baayen, R. H. (2001). Word frequency distributions (Vol. 18). Springer Science & Business Media. 10.1007/978-94-010-0844-0

[bib75] Bates, E., Bretherton, I. & Snyder, L. (1988). From first words to grammar: Individual differences and dissociable mechanisms. Cambridge University Press

[bib3] Bates, E., Bretherton, I., & Snyder, L. (1991). From first words to grammar: Individual differences and dissociable mechanisms (Vol. 20). Cambridge University Press.

[bib4] Bates, E., Marchman, V., Thal, D., Fenson, L., Dale, P., Reznick, J. S., Reilly, J., & Hartung, J. (1994). Developmental and stylistic variation in the composition of early vocabulary. Journal of Child Language, 21(1), 85–123. 10.1017/S0305000900008680, 8006096

[bib5] Bates, D., Mächler, M., Bolker, B., & Walker, S. (2015). Fitting linear mixed-effects models using lme4. Journal of Statistical Software, 67(1), 1–48. 10.18637/jss.v067.i01

[bib6] Bornstein, M. H., Cote, L. R., Maital, S., Painter, K., Park, S.-Y., Pascual, L., Pêcheux, M.-G., Ruel, J., Venuti, P., & Vyt, A. (2004). Cross-linguistic analysis of vocabulary in young children: Spanish, Dutch, French, Hebrew, Italian, Korean, and American English. Child Development, 75(4), 1115–1139. 10.1111/j.1467-8624.2004.00729.x, 15260868

[bib8] Braginsky, M., Yurovsky, D., Marchman, V. A., & Frank, M. C. (2019). Consistency and variability in children’s word learning across languages. Open Mind, 3, 52–67. 10.1162/opmi_a_00026, 31517175 PMC6716390

[bib10] Caselli, M. C., Bates, E., Casadio, P., Fenson, J., Fenson, L., Sanderl, L., & Weir, J. (1995). A cross-linguistic study of early lexical development. Cognitive Development, 10(2), 159–199. 10.1016/0885-2014(95)90008-X

[bib11] Caselli, C., Casadio, P., & Bates, E. (1999). A comparison of the transition from first words to grammar in English and Italian. Journal of Child Language, 26(1), 69–111. 10.1017/S0305000998003687, 10217890

[bib13] Davies, M. (2008). The Corpus of Contemporary American English (COCA): 560 million words, 1990–present. Available online at https://corpus.byu.edu/coca/.

[bib14] Fenson, L., Dale, P. S., Reznick, J. S., Bates, E., Thal, D. J., Pethick, S. J., Tomasello, M., Mervis, C. B., & Stiles, J. (1994). Variability in early communicative development. Monographs of the Society for Research in Child Development, 59(5), i–185. 10.2307/1166093, 7845413

[bib15] Fenson, L., Marchman, V. A., Thal, D. J., Dale, P. S., Reznick, J. S., & Bates, E. (2007). MacArthur-Bates Communicative Development Inventories: User’s guide and technical manual (2nd ed.). Brookes.

[bib16] Fernald, A., McRoberts, G. W., & Swingley, D. (2001). Infants’ developing competence in recognizing and understanding words in fluent speech. In Approaches to bootstrapping: Phonological, lexical, syntactic and neurophysiological aspects of early language acquisition (pp. 97–123). Benjamins. 10.1075/lald.23.08fer

[bib18] Frank, M. C., Braginsky, M., Yurovsky, D., & Marchman, V. A. (2017). Wordbank: An open repository for developmental vocabulary data. Journal of Child Language, 44(3), 677–694. 10.1017/S0305000916000209, 27189114

[bib19] Frank, M. C., Braginsky, M., Yurovsky, D., & Marchman, V. A. (2021). Variability and consistency in early language learning: The Wordbank project. MIT Press. 10.7551/mitpress/11577.001.0001

[bib22] Gentner, D. (1982). Why nouns are learned before verbs: Linguistic relativity versus natural partitioning. In S. Kuczaj (Ed.), Language development: Language, cognition and culture (pp. 301–334). Lawrence Erlbaum Associates Inc.

[bib21] Gentner, D., Boroditsky, L., Bowerman, M., & Levinson, S. (2001). Individuation, relativity, and early word learning. In M. Bowerman & S. Levinson (Eds.), Language acquisition and conceptual development (pp. 215–256). Cambridge University Press. 10.1017/CBO9780511620669.010

[bib24] Gillette, J., Gleitman, H., Gleitman, L., & Lederer, A. (1999). Human simulations of vocabulary learning. Cognition, 73(2), 135–176. 10.1016/S0010-0277(99)00036-0, 10580161

[bib25] Gleitman, L. R., & Gleitman, H. (1992). A picture is worth a thousand words, but that’s the problem: The role of syntax in vocabulary acquisition. Current Directions in Psychological Science, 1(1), 31–35. 10.1111/1467-8721.ep10767853

[bib76] Goldin-Meadow, S., Seligman, M. E. P., & Gelman, R. (1976). Language in the two-year old. Cognition, 4(2), 189–202. 10.1016/0010-0277(76)90004-4

[bib26] Golinkoff, R. M., Shuff-Bailey, M., Olguin, R., & Ruan, W. (1995). Young children extend novel words at the basic level: Evidence for the principle of categorical scope. Developmental Psychology, 31(3), 494–507. 10.1037/0012-1649.31.3.494

[bib77] Goodman, J. C., Dale, P. S., & Li, P. (2008). Does frequency count? Parental input and the acquisition of vocabulary. Journal of Child Language, 35(3), 515–531. 10.1017/S0305000907008641, 18588713

[bib78] Greenfield, P. M. (1978). How much is one word? Journal of Child Language, 5(2), 347–352. 10.1017/S0305000900007522

[bib27] Hansen, P. (2017). What makes a word easy to acquire? The effects of word class, frequency, imageability and phonological neighbourhood density on lexical development. First Language, 37(2), 205–225. 10.1177/0142723716679956

[bib28] Hao, M., Liu, Y., Shu, H., Xing, A., Jiang, Y., & Li, P. (2015). Developmental changes in the early child lexicon in Mandarin Chinese. Journal of Child Language, 42(3), 505–537. 10.1017/S030500091400018X, 24967509

[bib79] Hart, B., & Risley, T. R. (1995). Meaningful differences in the everyday experience of young American children. P. H. Brookes.

[bib29] Kauschke, C., & Hofmeister, C. (2002). Early lexical development in German: A study on vocabulary growth and vocabulary composition during the second and third year of life. Journal of Child Language, 29(4), 735–757. 10.1017/S0305000902005330, 12471971

[bib30] Kim, M., McGregor, K. K., & Thompson, C. K. (2000). Early lexical development in English- and Korean-speaking children: Language-general and language-specific patterns. Journal of Child Language, 27(2), 225–254. 10.1017/S0305000900004104, 10967886

[bib31] Kousta, S.-T., Vigliocco, G., Vinson, D. P., Andrews, M., & Del Campo, E. (2011). The representation of abstract words: Why emotion matters. Journal of Experimental Psychology: General, 140(1), 14–34. 10.1037/a0021446, 21171803

[bib32] Landau, B., & Gleitman, L. R. (1985). Language and experience: Evidence from the blind child (Vol. 8). Harvard University Press.

[bib33] Landau, B., Smith, L. B., & Jones, S. S. (1988). The importance of shape in early lexical learning. Cognitive Development, 3(3), 299–321. 10.1016/0885-2014(88)90014-7

[bib80] Lee, J. N., & Naigles, L. R. (2005). The input to verb learning in Mandarin Chinese: A role for syntactic bootstrapping. Developmental Psychology, 41(3), 529–540. 10.1037/0012-1649.41.3.529, 15910160

[bib34] MacWhinney, B. (2000). The CHILDES project: Tools for analyzing talk. Erlbaum. 10.4324/9781315805641

[bib36] Ma, W., Golinkoff, R. M., Hirsh-Pasek, K., McDonough, C., & Tardif, T. (2009). Imageability predicts the age of acquisition of verbs in Chinese children. Journal of Child Language, 36(2), 405–423. 10.1017/S0305000908009008, 18937878 PMC2925137

[bib38] Mani, N., & Ackermann, L. (2018). Why do children learn the words they do? Child Development Perspectives, 12(4), 253–257. 10.1111/cdep.12295

[bib39] Markman, E. M. (1990). Constraints children place on word meanings. Cognitive Science, 14(1), 57–77. 10.1207/s15516709cog1401_4

[bib40] McDonough, C., Song, L., Hirsh-Pasek, K., Golinkoff, R. M., & Lannon, R. (2011). An image is worth a thousand words: Why nouns tend to dominate verbs in early word learning. Developmental Science, 14(2), 181–189. 10.1111/j.1467-7687.2010.00968.x, 21359165 PMC3043374

[bib81] Medina, T. N., Snedeker, J., Trueswell, J. C., & Gleitman, L. R. (2011). How words can and cannot be learned by observation. Proceedings of the National Academy of Sciences, 108(22), 9014–9019. 10.1073/pnas.1105040108, 21576483 PMC3107260

[bib43] Naigles, L. R., & Hoff-Ginsberg, E. (1998). Why are some verbs learned before other verbs? Effects of input frequency and structure on children’s early verb use. Journal of Child Language, 25(1), 95–120. 10.1017/S0305000997003358, 9604570

[bib44] Nelder, J. A., & Mead, R. (1965). A simplex method for function minimization. Computer Journal, 7, 308–313. 10.1093/comjnl/7.4.308

[bib45] Nice, M. M. (1925). Length of sentences as a criterion of a child’s progress in speech. Journal of Educational Psychology, 16(6), 370–379. 10.1037/h0073259

[bib46] Ogura, T., Dale, P. S., Yamashita, Y., Murase, T., & Mahieu, A. (2006). The use of nouns and verbs by Japanese children and their caregivers in book-reading and toy-playing contexts. Journal of Child Language, 33(1), 1–29. 10.1017/S0305000905007270, 16566318

[bib47] Oshima-Takane, Y., Goodz, E., & Derevesky, J. L. (1996). Birth order effects on early language development: Do secondborn children learn from overheard speech? Child Development 67, 621–634. 10.1111/j.1467-8624.1996.tb01755.x

[bib48] Paivio, A., Yuille, J. C., & Madigan, S. A. (1968). Concreteness, imagery, and meaningfulness values for 925 nouns. Journal of Experimental Psychology, 76(1p2), 1–25. 10.1037/h0025327, 5672258

[bib50] Papaeliou, C. F., & Rescorla, L. A. (2011). Vocabulary development in Greek children: A cross-linguistic comparison using the Language Development Survey. Journal of Child Language, 38(4), 861–887. 10.1017/S030500091000053X, 21729371

[bib51] Peters, A. M. (1985). Language segmentation: Operating principles for the perception and analysis of language. In D. I. Slobin (Ed.), The crosslinguistic study of language acquisition Vol 2: Theoretical issues (pp. 1029–1067). Lawrence Erlbaum Associates Inc.

[bib52] Pine, J. M. (1995). Variation in vocabulary development as a function of birth order. Child Development, 66(1), 272–281. 10.1111/j.1467-8624.1995.tb00870.x

[bib54] R Core Team. (2017). R: A language and environment for statistical computing. R Foundation for Statistical Computing. https://www.R-project.org/

[bib55] Robinson, B. F., & Mervis, C. B. (1999). Comparing productive vocabulary measures from the CDI and a systematic diary study. Journal of Child Language, 26(1), 177–185. 10.1017/S0305000998003663, 10217894

[bib56] Sandhofer, C. M., Smith, L. B., & Luo, J. (2000). Counting nouns and verbs in the input: Differential frequencies, different kinds of learning? Journal of Child Language, 27(3), 561–585. 10.1017/S0305000900004256, 11089339

[bib57] Schults, A., Tulviste, T., & Konstabel, K. (2012). Early vocabulary and gestures in Estonian children. Journal of Child Language, 39(3), 664–686. 10.1017/S0305000911000225, 21878148

[bib58] Scott, G. G., Keitel, A., Becirspahic, M., Yao, B., & Sereno, S. C. (2019). The Glasgow Norms: Ratings of 5,500 words on nine scales. Behavior Research Methods, 51(3), 1258–1270. 10.3758/s13428-018-1099-3, 30206797 PMC6538586

[bib59] Slobin, D. I. (1985). Crosslinguistic evidence for the language-making capacity. In D. I. Slobin (Ed.), The crosslinguistic study of language acquisition Vol 2: Theoretical issues (pp. 1157–1249). Lawrence Erlbaum Associates Inc.

[bib60] Smith, L. B., Jones, S. S., Landau, B., Gershkoff-Stowe, L., & Samuelson, L. (2002). Object name learning provides on-the-job training for attention. Psychological Science, 13(1), 13–19. 10.1111/1467-9280.00403, 11892773

[bib82] Smith, L., & Yu, C. (2008). Infants rapidly learn word-referent mappings via cross-situational statistics. Cognition, 106(3), 1558–1568. 10.1016/j.cognition.2007.06.010, 17692305 PMC2271000

[bib61] Smolík, F. (2019). Imageability and neighborhood density facilitate the age of word acquisition in Czech. Journal of Speech, Language, and Hearing Research, 62(5), 1403–1415. 10.1044/2018_JSLHR-L-18-0242, 31046539

[bib62] Snedeker, J., & Gleitman, L. R. (2004). Why it is hard to label our concepts. In D. Geoffrey Hall & S. R. Waxman (Eds.), Weaving a lexicon (pp. 257–294). MIT Press. 10.7551/mitpress/7185.003.0012

[bib63] Snedeker, J., Geren, J., & Shafto, C. L. (2007). Starting over: International adoption as a natural experiment in language development. Psychological Science, 18(1), 79–87. 10.1111/j.1467-9280.2007.01852.x, 17362382

[bib64] Snedeker, J., Geren, J., & Shafto, C. L. (2012). Disentangling the effects of cognitive development and linguistic expertise: A longitudinal study of the acquisition of English in internationally-adopted children. Cognitive Psychology, 65(1), 39–76. 10.1016/j.cogpsych.2012.01.004, 22417632

[bib65] Sundara, M. (2018). Why do children pay more attention to grammatical morphemes at the ends of sentences? Journal of Child Language, 45(3), 703–716. 10.1017/S0305000917000356, 29067896

[bib66] Swingley, D., & Humphrey, C. (2018). Quantitative linguistic predictors of infants’ learning of specific English words. Child Development, 89(4), 1247–1267. 10.1111/cdev.12731, 28146333 PMC5538897

[bib83] Tardif, T., Gelman, S. A., & Xu, F. (1999). Putting the “noun bias” in context: A comparison of English and Mandarin. Child Development, 70(3), 620–635. 10.1111/1467-8624.00045

[bib68] Trueswell, J. C., Medina, T. N., Hafri, A., & Gleitman, L. R. (2013). Propose but verify: Fast mapping meets cross-situational word learning. Cognitive Psychology, 66(1), 126–156. 10.1016/j.cogpsych.2012.10.001, 23142693 PMC3529979

[bib69] Wallentin, M., & Trecca, F. (2022). Vocabulary content rather than size predicts sex/gender before the age of three years. In Proceedings of the 8th Conference of the Scandinavian Association for Language and Cognition.

[bib70] Xu, F. (2002). The role of language in acquiring object kind concepts in infancy. Cognition, 85(3), 223–250. 10.1016/S0010-0277(02)00109-9, 12169410

[bib71] Yu, C., & Smith, L. B. (2007). Rapid word learning under uncertainty via cross-situational statistics. Psychological Science, 18(5), 414–420. 10.1111/j.1467-9280.2007.01915.x, 17576281

