# Supplementary material for: It’s All in the Interaction: Early Acquired Words Are Both Frequent and Highly Imageable
Source: Open Mind (Camb). 2024 Mar 26;8:309–32. doi: 10.1162/opmi_a_00130 (PMC10990573; doi:10.1162/opmi_a_00130)
Supplement: Supplementary file 1 [file opmi-08-309-s001.docx]

**Supplementary Materials**

**Table S1**

Stepwise fit comparisons for logistic mixed effects regression models *with* age interaction. Predictors are added to each successive model and compared for fit using likelihood ratio testing (16-20 months).

|  | Base | Model 1 | Model 2 | Model 3 | Model 4 |
| --- | --- | --- | --- | --- | --- |
| Intercept | -2.98*** | -2.98*** | -2.98*** | -2.78*** | -5.17*** |
| (Std. Err) | (0.12) | (0.11) | (0.11) | (0.11) | (0.24) |
| Age | 0.79*** | 0.79*** | 0.79*** | 0.79*** | 0.79*** |
|  | (0.09) | (0.09) | (0.09) | (0.09) | (0.09) |
| Imageability |  | 0.79*** | 1.19*** | 1.03*** | 0.11 |
|  |  | (0.07) | (0.08) | (0.08) | (0.12) |
| Frequency |  |  | 0.84*** (0.08) | 0.9*** (0.07) | 1.08*** (0.07) |
| Frequency x Imageability |  |  |  | 0.4*** (0.06) | 0.34*** (0.06) |
| Predicate |  |  |  |  | 1.58*** (0.22) |
| Noun |  |  |  |  | 3.25*** (0.31) |
| Social |  |  |  |  | 4.02*** (0.3) |
| Improved Fit? |  | Yes | Yes | Yes | Yes |
| ANOVA ChiSq |  | 109.86*** | 112.33*** | 37.9*** | 170.03*** |

Best fit at each step bolded. Best fit overall boxed.

’ *p <* 0*.*1, * *p <* 0*.*05, ** *p <* 0*.*01, *** *p <* 0*.*001

**Table S2**

Stepwise fit comparisons for logistic mixed effects regression models *with* age interaction. Predictors are added to each successive model and compared for fit using likelihood ratio testing (21-25 months).

|  | Base | Model 1 | Model 2 | Model 3 | Model 4 |
| --- | --- | --- | --- | --- | --- |
| Intercept | -0.65*** | -0.65*** | -0.65*** | -0.47*** | -2.55*** |
| (Std. Err) | (0.11) | (0.11) | (0.11) | (0.11) | (0.22) |
| Age | 0.54*** | 0.54*** | 0.54*** | 0.54*** | 0.54*** |
|  | (0.09) | (0.09) | (0.09) | (0.09) | (0.09) |
| Imageability |  | 0.69*** | 1.1*** | 0.97*** | 0.19 |
|  |  | (0.06) | (0.07) | (0.07) | (0.1) |
| Frequency |  |  | 0.85*** (0.07) | 0.92*** (0.07) | 1.06*** (0.06) |
| Frequency x Imageability |  |  |  | 0.37*** (0.06) | 0.3*** (0.06) |
| Predicate |  |  |  |  | 1.52*** (0.2) |
| Noun |  |  |  |  | 2.81*** (0.28) |
| Social |  |  |  |  | 3.24*** (0.26) |
| Improved Fit? |  | Yes | Yes | Yes | Yes |
| ANOVA ChiSq |  | 104.74*** | 147.74*** | 40.65*** | 136.51*** |

Best fit at each step bolded. Best fit overall boxed.

’ *p <* 0*.*1, * *p <* 0*.*05, ** *p <* 0*.*01, *** *p <* 0*.*001

**Table S3**

Stepwise fit comparisons for logistic mixed effects regression models *with* age interaction. Predictors are added to each successive model and compared for fit using likelihood ratio testing (26-30 months).

|  | Base | Model 1 | Model 2 | Model 3 | Model 4 |
| --- | --- | --- | --- | --- | --- |
| Intercept | 1.00*** | 1.00*** | 1.00*** | 1.16*** | -0.73*** |
| (Std. Err) | (0.11) | (0.11) | (0.11) | (0.11) | (0.22) |
| Age | 0.48*** | 0.48*** | 0.48*** | 0.48*** | 0.48*** |
|  | (0.09) | (0.09) | (0.09) | (0.09) | (0.09) |
| Imageability |  | 0.62*** | 1.04*** | 0.93*** | 0.24* |
|  |  | (0.06) | (0.06) | (0.06) | (0.1) |
| Frequency |  |  | 0.86*** (0.06) | 0.91*** (0.06) | 1.04*** (0.06) |
| Frequency x Imageability |  |  |  | 0.31*** (0.05) | 0.25*** (0.05) |
| Predicate |  |  |  |  | 1.5*** (0.19) |
| Noun |  |  |  |  | 2.52*** (0.27) |
| Social |  |  |  |  | 2.69*** (0.25) |
| Improved Fit? |  | Yes | Yes | Yes | Yes |
| ANOVA ChiSq |  | 100.91*** | 177.97*** | 36.1*** | 113.39*** |

Best fit at each step bolded. Best fit overall boxed.

’ *p <* 0*.*1, * *p <* 0*.*05, ** *p <* 0*.*01, *** *p <* 0*.*001

**Figure S1**

Proportion of children’s vocabulary by syntactic category relative to their total vocabulary size.


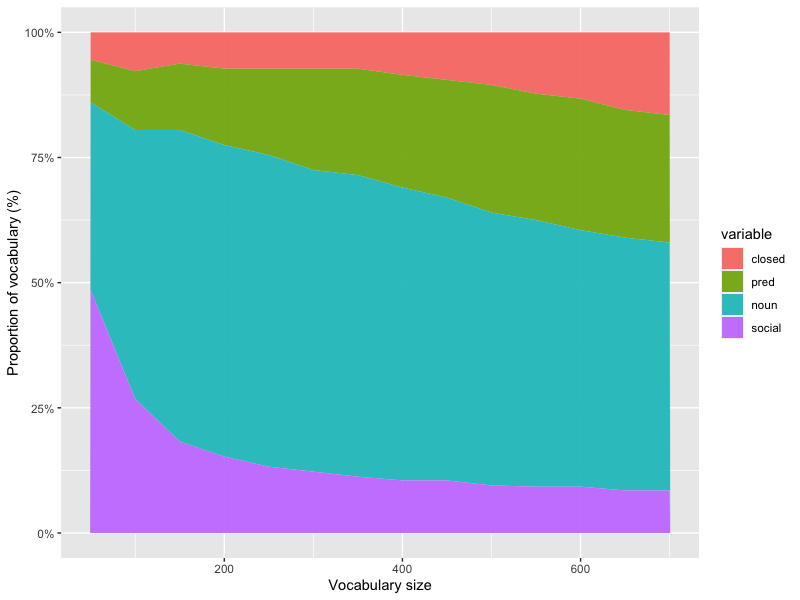


**Figure S2**

Density plots tracking the proportion of words by syntactic category at each possible value for a given predictor: imageability (top), log frequency (middle), and their interaction (bottom).

**
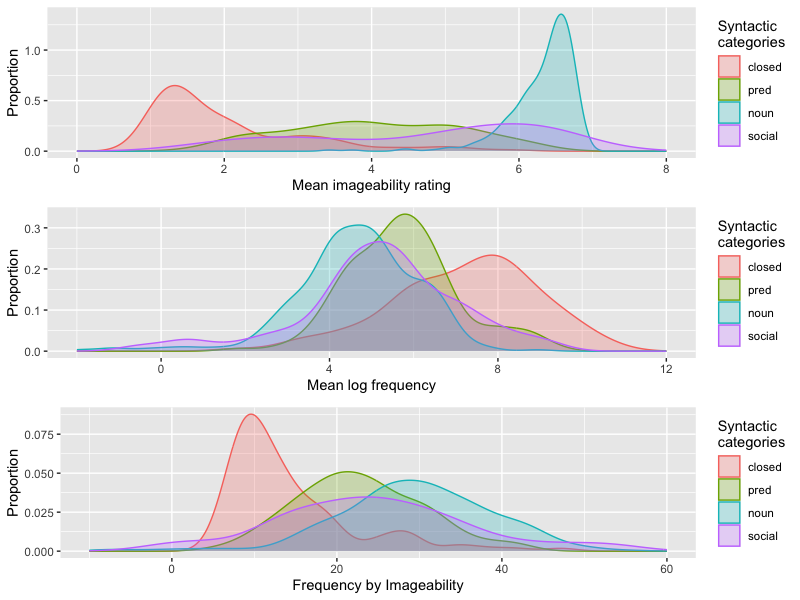
**

**Figure S3**

Comparison of effect sizes for our age interaction model across different optimizers constructed using *allFit* and *plot.fixef.allFit* (Bernabeu, 2022)


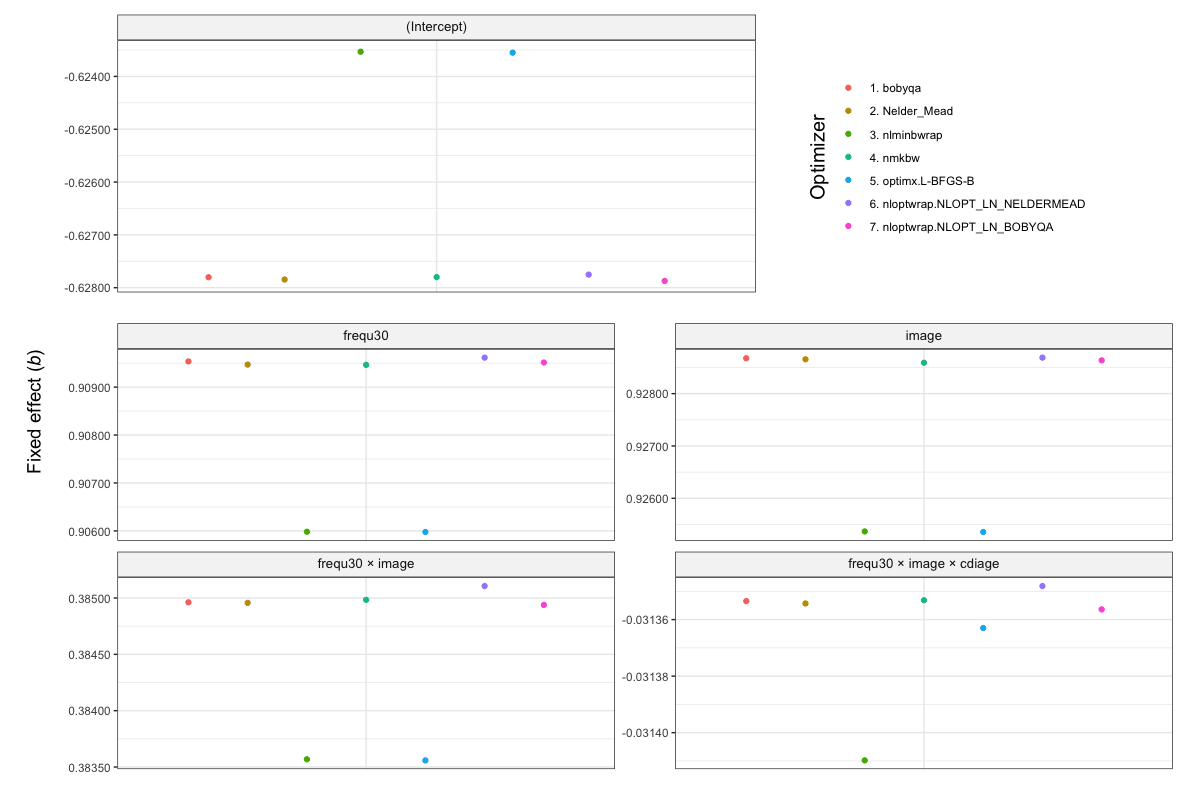


Bernabeu, P. (2022). Language and sensorimotor simulation in conceptual processing: Multilevel analysis and statistical power. Lancaster University. <https://doi.org/10.17635/lancaster/thesis/1795>
